# Efficacy of stimulants for preschool attention‐deficit/hyperactivity disorder: A systematic review and meta‐analysis

**DOI:** 10.1002/jcv2.12146

**Published:** 2023-02-25

**Authors:** Luisa S. Sugaya, Luis C. Farhat, Pietro Califano, Guilherme V. Polanczyk

**Affiliations:** ^1^ Department of Psychiatry Faculdade de Medicina FMUSP Universidade de São Paulo São Paulo Brazil; ^2^ National Institute of Developmental Psychiatry (INPD) CNPq São Paulo Brazil

**Keywords:** attention‐deficit hyperactivity disorder, lisdexamfetamine, meta‐analysis, methylphenidate, preschool, stimulants

## Abstract

**Background:**

Robust synthesis of evidence to support treatment recommendations for preschoolers with attention‐deficit/hyperactivity disorder (ADHD) is lacking. The aim of this systematic review and meta‐analysis was to review currently available evidence to evaluate the efficacy and acceptability of stimulants for preschool children with ADHD.

**Methods:**

We searched electronic databases (CENTRAL, Embase, PubMed) from the database inception to March, 2022; and clinical trial registries through WHO ICTRP from the database inception to July, 2022, and selected double‐blinded randomized controlled trials (RCTs) that compared stimulants against placebo for the treatment of preschoolers (age ≤ 7 years) with ADHD. Change in ADHD symptom severity was the primary outcome (efficacy) and all‐cause dropout rates (acceptability) was the secondary outcome. Data were pooled with random‐effects models weighted by the inverse of the variance. Risk of bias of individual studies were assessed with the Cochrane Risk of Bias tool version 2. The Grading of Recommendations Assessment, Development, and Evaluation approach was used to assess the quality of evidence. This study is registered with PROSPERO (CRD42022348597).

**Results:**

Five RCTs (three methylphenidate immediate‐release, one methylphenidate extended‐release, and one lisdexamfetamine) were included. The analysis of efficacy was based on 489 participants. Meta‐analysis of change in ADHD symptom severity demonstrated a significant effect in favor of stimulants over placebo (standardized mean difference = −0.59; 95% CI −0.77, −0.41; *p* < 0.0001). There was no evidence of heterogeneity but some concerns about publication bias. Regardless, the confidence of evidence was considered moderate. For acceptability, stimulants did not lead to an increased rate of all‐cause discontinuation rates in comparison to placebo (OR = 0.59; 95% CI 0.15, 2.37; *p* = 0.45) but the confidence of estimate was very low.

**Conclusions:**

Our findings demonstrated that stimulants are efficacious in reducing ADHD symptoms among preschool children. Clinicians should consider the use of stimulants when making treatment recommendations for preschoolers with ADHD.

## Graphical abstract

•This is the first meta‐analysis to evaluate the efficacy and acceptability of stimulants for the treatment of preschool children with ADHD. •Our findings demonstrated that stimulants are effective in reducing ADHD symptoms among preschool children (SMD = −0.59; 95% CI −0.77, −0.41; *p* < 0.0001) with moderate confidence in estimates. •In clinical practice, stimulants should be considered by clinicians for the treatment of preschoolers with ADHD.

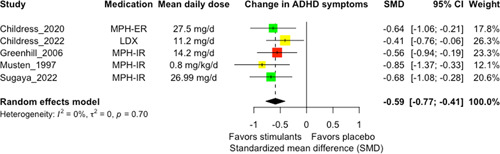

**Key points**

- This is the first meta‐analysis to evaluate the efficacy and acceptability of stimulants for the treatment of preschool children with attention‐deficit/hyperactivity disorder (ADHD).
- Five randomized controlled trials (RCTs) were included in the analysis. Our findings demonstrated that stimulants are effective in reducing ADHD symptoms among preschoolers (standardized mean difference = −0.59; 95% CI −0.77, −0.41; *p* < 0.0001) with moderate confidence in estimates.
- Additional RCTs evaluating the short‐ and long‐term efficacy and safety of simulants for preschool children are still a priority in the field. Future research should fill evidence gaps including the effect of these medications on functional outcomes as well potential moderators and mediators of increased/decreased effect.
- In clinical practice, stimulants should be considered by clinicians for the treatment of preschoolers with ADHD.

## INTRODUCTION

Attention deficit/hyperactivity disorder (ADHD) is a common neurodevelopmental disorder characterized by symptoms of inattention, hyperactivity, and/or impulsivity that typically emerge in early childhood (Posner et al., 2020). Preschool ADHD is associated with significant impairment, including peer rejection, academic underachievement, and increased risk of morbidity and mortality (Dalsgaard et al., 2015a, 2015b; Spira & Fischel, 2005). Clinical diagnosis of ADHD persists from preschool to school‐age periods in approximately 90% of children (Riddle et al., 2013) and is associated with concurrent comorbidities, such as neurodevelopmental disorders, and future onset of new disorders, such as depression (Posner et al., 2020).

The prevalence of ADHD among preschoolers is estimated at 2.7%–4.3% (Vasileva et al., 2021). The clinical assessment of preschool children represents a challenge to clinicians, who must consider contextual factors, the presence of other neurodevelopmental conditions, and variations of normal development. A precise diagnosis of ADHD in preschool years represents an opportunity for early interventions, which may potentially alter the trajectory of the disorder and prevent the cascade of increasing symptoms, impairment, and comorbidity (Ruiz‐Goikoetxea et al., 2018).

In the literature, there is a limited number of studies evaluating pharmacological treatments for preschoolers with ADHD. The Preschool ADHD Treatment Study (PATS) (Greenhill et al., 2006), a multi‐center, multi‐phase randomized controlled trial (RCT) evaluating the safety and efficacy of methylphenidate in children aged 3–5.5 years with ADHD has been considered the best evidence in the field. In the PATS, a significant improvement in ADHD symptoms was observed with methylphenidate immediate‐release compared to placebo, but effect sizes were smaller and discontinuation rates were higher than previously reported in school‐age children (Greenhill et al., 2006). Thus, current guidelines recommend medication for children who remain symptomatic and impaired after a trial of behavioral parent training (BPT) (Cortese, 2020). Nevertheless, meta‐analyses have shown that BPT may lead to a reduction in ADHD symptoms only when outcomes are reported by unblinded parents (Rimestad et al., 2019). Also, barriers to the implementation of BPT (e.g., trained professionals' availability) may limit access to treatment. Indeed, in the US, most preschoolers with ADHD receive medications with or without psychosocial treatments; only approximately 15% receive behavioral intervention exclusively; and part of the children receive neither treatment (Visser et al., 2016).

In the past 2 years, new RCTs evaluating stimulants for preschool ADHD have been published, providing more evidence to inform the field (Childress et al., 2020, 2022; Sugaya et al., 2022). Systematic reviews and meta‐analyses have supported the use of stimulants as a first‐line treatment for school‐aged children and adolescents (Cortese et al., 2018). However, to date, there is no meta‐analysis evaluating the effects of stimulants on the treatment of preschoolers with ADHD. To fill this gap, we conducted a systematic review and meta‐analysis using data from double‐blind RCTs evaluating the effects of stimulants in comparison to placebo. Efficacy measured as change in ADHD symptoms was the primary outcome and acceptability measured as all‐cause dropout rates was the secondary outcome. In addition, we provide a discussion integrating research findings and clinical practice.

## METHODS

### Eligibility criteria

In this systematic review and meta‐analysis, we included double‐blinded RCTs that enrolled preschool children (age ≤ 7 years) with a primary diagnosis of ADHD according to the Diagnostic and Statistical Manual of Mental Disorders (DSM‐III/III‐R/IV/IV‐TR/5), or equivalent hyperkinetic disorder according to ICD‐9/10 criteria; administered stimulants against placebo; and assessed change in ADHD symptom severity as an outcome. We did not restrict eligibility based on ADHD presentation, psychiatric or neurological comorbidities, IQ status, sex, race/ethnicity, or other sociodemographic characteristics. We included any stimulant, either methylphenidate or amphetamines, irrespective of formulation and dosages. We did not restrict eligibility based on dosing design, treatment duration, or previous/co‐occurring psychotherapeutic treatments. Both parallel and crossover RCTs were eligible for inclusion. We put no restrictions on language or publication year.

We excluded studies that recruited participants who did not have a formal ADHD diagnosis or who had minimal brain dysfunction or ADHD diagnosis secondary to a genetic syndrome or perinatal exposure. We excluded studies that administered other pharmacological agents in addition to stimulants. Quasi‐RCTs (e.g., use of Latin square without mention of random*; alternate days of the week) and N‐of‐1 trials were excluded. The protocol of the study was pre‐registered in PROSPERO (CRD42022348597).

### Search strategy

We searched Embase and PubMed from the database inception to March 24th, 2022, and the Cochrane Central Register of Controlled Trials and the WHO ICTRP from the database inception to July 23rd, 2022. The search terms were tailored to each database and are provided in the Supporting Information S1. We also hand‐searched ClinicalTrials.gov as well as references of previous systematic reviews to look for additional studies. We emailed authors to gather unpublished data.

### Study selection and data extraction

All stages of study selection and data extraction were conducted independently by a pair of researchers (LCF, PC). Any discrepancies were double‐checked and resolved by discussion with another member of the review team (GVP).

Records identified through searchers in electronic databases were initially managed in EndNote to remove duplicates. Then, titles and abstracts were reviewed in Rayyan to screen potentially eligible records. The full text, supplementary materials, and other data sources were then retrieved from potentially eligible records to fully assess their eligibility and determine inclusion or exclusion. Online software was used to translate records if needed. Data from multiple reports of the same study were linked together.

We extracted summary outcome data at the study endpoint from the included RCTs. We also coded the following pieces of information: trial characteristics, including recruiting area, year study started, sponsorship (industry/non‐industry), design (parallel/crossover), and number of participating centers (single/multi‐center); participant characteristics, including age (range, mean [SD]), gender (proportion of boys), race (proportion of Asian‐American/Black/Indian‐American/White), ethnicity (proportion of Hispanics/Latinos), and diagnostic criteria; treatment characteristics, including duration, requirement of previous/concomitant psychotherapeutic interventions, medication administered, dosages (minimum, maximum, and mean). For fixed‐dose RCTs which randomized participants to multiple doses of the same medication, we pooled the arms of the same medication at different doses provided that the doses were judged to be therapeutic. For crossover RCTs, we preferred pre‐crossover data to avoid unit‐of‐analysis problems. However, most crossover RCTs do not provide pre‐crossover data nor provide a comprehensive report of data to allow their inclusion appropriately, considering the within‐subject design. Hence, to avoid excluding eligible studies, when pre‐crossover data was not available, we extracted data from crossover RCTs as parallel RCTs.

The primary outcome was efficacy, measured as change in ADHD symptom severity. When multiple rating scales were provided, we preferred the following, in this order: the ADHD rating scale (ADHD‐RS) (DuPaul, 1998) the Swanson, Nolan, and Pelham ADHD rating scale (SNAP) (Bussing et al., 2008), and the Conner's Parent/Teacher rating scales (CPRS/CTRS) (Conners et al., 1998a, 1998b). However, we also extracted data based on any other rating scales if those were the only data reported. When multiple informants were provided, we preferred the following, in this order: clinician, parent‐teacher combined, teacher, and parent. Change from baseline scores was preferred over endpoint scores. Intention‐to‐treat (ITT) analyses were preferred, and we used the method adopted by the study to handle missing data, usually mixed model repeated measures or last observation carried forward. However, we also extracted data based on participants who completed the study (or modified ITT) if that was the only analysis reported. We extracted published standard deviations when available, but for some studies they were not reported. In those cases, we looked for 95% confidence intervals (CI), standard errors, t‐values, or *p*‐values which could be used to calculate standard deviations as reported in the Cochrane Handbook. We did not impute SD for any of the studies. The secondary outcome was acceptability, measured as the all‐cause dropout rates. Of note, in the pre‐specified protocol, we also specified our interest to examine tolerability, measured as adverse events dropout rates. However, only one study reported data for this outcome.

### Data analysis

Standardized mean difference (SMD) was used as the effect size index for efficacy and odds ratio (OR) was used for acceptability. We pooled data with random‐effects models weighted by the inverse of the variance. The Q‐test and the *I* ^2^ statistic were used to evaluate heterogeneity. In accordance with the pre‐specified protocol, we stratified analyses by compound and informants in subgroup analyses. Inferences on subgroup differences were made considering the Q‐test for between subgroups heterogeneity rather than statistical significance within subgroups.

The robustness of our findings was examined in sensitivity analyses performed by excluding studies rated at high risk of bias, crossover studies without pre‐crossover data, studies with less than 1 week of treatment duration, and studies that required participants to receive BPT or other psychotherapeutic intervention prior to pharmacological treatment.

For efficacy, we assessed the risk of bias of RCTs with the Cochrane tool (Sterne et al., 2019). To assess publication bias, we created funnel plots to assess asymmetry and we used the Egger regression test to evaluate small‐study effects. If asymmetry was detected, we calculated adjusted effect sizes considering the trim‐and‐fill method. The Grading of Recommendations Assessment, Development, and Evaluation (GRADE) approach was applied to assess the quality of evidence for efficacy and acceptability. We only considered the five factors that determine downgrading of the quality of evidence. The presence of each factor downgraded evidence one or two levels and reductions were added together to reduce the quality of evidence for the outcome.

All analyses were done in R with package *meta* (version 4.19–2) (Balduzzi et al., 2019). This study was conducted according to the Cochrane Handbook and reported according to the Preferred Reporting Items for Systematic Reviews and Meta‐Analysis (PRISMA) recommendations. A PRISMA checklist was provided in the Supporting Information S1.

## RESULTS

### Study selection and characteristics

**FIGURE 1:**
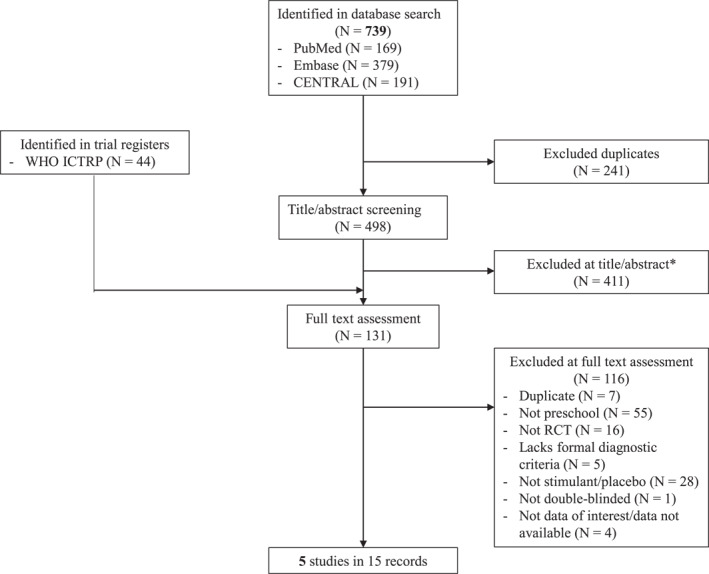
Study selection procedures.

We retrieved 739 records through database search and screened 498 records after excluding duplicates. Of these, 87 records were selected for further assessment, along with 44 records identified in trial registers. Of the 131 records inspected in detail, 116 were excluded and 15 representing 5 RCTs were included (Figure 1).

The five RCTs involved 489 participants whose mean age (SD) was 4.95 (0.61) years and of which 366 (75%) were males. Most studies were conducted in the United States (3, 60%), were not sponsored by industry (3, 60%), and adopted a parallel design (4, 80%). Methylphenidate immediate‐release (3, 60%), methylphenidate extended‐release (1, 20%), and lisdexamfetamine (1, 20%) were the stimulants administered against placebo. Treatment trials lasted for a median of 4 weeks (IQR 2–6) (Table 1). One (20%), 2 (40%), and 2 (40%) studies were rated at high, moderate, and low risk of bias, respectively. Domain‐level risk of bias assessments for each study were provided in the Supporting Information S1.

**TABLE 1:** Characteristics of included studies.

	Country (year of study)	Number of participants	Age range, years (mean)	Proportion of males, %	Medication	Mean dose	Min‐max dose	Dosing schedule	Treatment duration, week	Design	Instrument	Informant	Funding
Childress_2020	United States (2016)	90	4–5.7 (4.9)	76	Methylphenidate extended‐release	27.5 mg/d	10–40 mg/d	Flexible	2	Parallel	ADHD‐RS‐IV	Clinician	Rhodes
Childress_2022	United Sates (2017)	152	4–5 (5.11)	67	Lisdexamfetamine	11.2 mg/d	10–30 mg/d	Fixed	6	Parallel	ADHD‐RS‐IV‐PS‐	Clinician	Shire
Greenhill_2006	United States (2001)	114	3–5.5 (4.76)	75	Methylphenidate immediate‐release	14.2 mg/d	3.75–22.5 mg/d	Fixed	4	Parallel	SNAP‐IV	Parent‐teacher combined	Non‐industry
Musten_1997	Canada (NR)	31	4–6 (4.84)	84	Methylphenidate immediate‐release	0.8 mg/kg/d	0.6–1 mg/kg/d	Fixed	∼1	Crossover	CPRS‐R	Parent	Non‐industry
Sugaya_2022	Brazil (2016)	153	3.9–5.9 (5.0)	83	Methylphenidate immediate‐release	1.23 mg/kg/d	1.0–1.25 mg/kg/d	Flexible	8	Parallel	SNAP‐IV	Parent‐teacher combined	Non‐industry

Abbreviation: NR, Not reported.

### Efficacy

**FIGURE 2:**
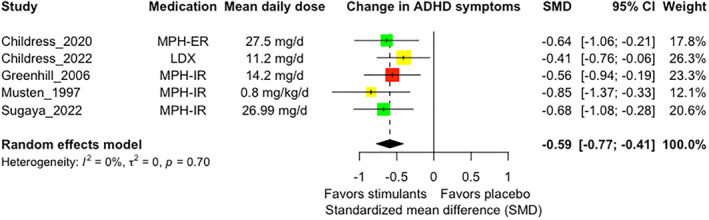
Forest plot for change in ADHD symptom severity. Risk of bias: green, low risk; yellow, some concern; red, high risk. Abbreviations: ADHD, attention‐deficit/hyperactivity disorder; LDX, lisdexamfetamine; MPH‐ER, methylphenidate extended‐release; MPH‐IR, methylphenidate immediate‐release; SMD, standardized mean difference.

Meta‐analysis of change in ADHD symptom severity demonstrated a significant effect in favor of stimulants over placebo (SMD = −0.59; 95% CI −0.77, −0.41; *p* < 0.0001) (Figure 2). There was no evidence of heterogeneity (*Q* = 2.18, *p* = 0.70; *I* ^2^ 0%). Test for subgroups did not identify significant differences between methylphenidate versus amphetamine (*Q* = 1.37, *p* = 0.24) nor between clinician, parent‐teacher combined, and parent outcomes (*Q* = 1.32, *p* = 0.52).

These findings did not change after excluding studies rated at high risk of bias (Figure S1), with less than 2 weeks of treatment duration (Figure S2), which required participants to receive psychotherapy prior to pharmacological treatment (Figure S3), and crossover studies (Figure S4).

Despite including less than 10 trials, the funnel plot indicated slight asymmetry at visual inspection (Figure S5), and the Egger regression test was significant (*t* = −3.73, *p* = 0.03). Adjusted effect sizes with the trim‐and‐fill method remained significant despite the addition of 2 studies (SMD = −0.52, 95% CI −0.68, −0.36, *p* < 0.0001). Still, we considered that the meta‐analysis provided moderate quality of evidence for this outcome. Domain‐level quality of evidence assessments were provided in the Supporting Information S1.

### Acceptability

**FIGURE 3:**
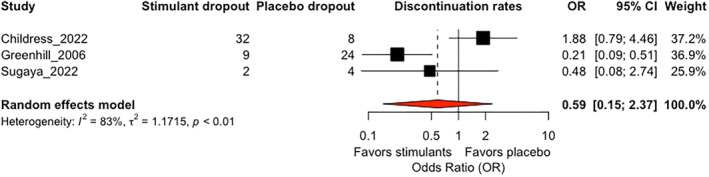
Forest plot for discontinuation rate.

Meta‐analysis of total discontinuation rates demonstrated a non‐significant effect (OR = 0.59; 95% CI 0.15, 2.37; *p* = 0.45) (Figure 3). There was evidence of heterogeneity (*Q* = 12.09, *p* = 0.002; *I* ^2^ 83.5%). Test for subgroups identified significant differences between methylphenidate versus amphetamine (*Q* = 11.40, *p* = 0.0007) and a significant effect was found for methylphenidate (OR = 0.25; 95% CI 0.11, 0.54; *p* = 0.0005; *I* ^2^ 0%), but not for amphetamines (OR = 1.88; 95% CI 0.79, 4.46; *p* = 0.15). We considered that the meta‐analysis provided very low quality of evidence for this outcome. Domain‐level quality of evidence assessments were provided in the Supporting Information S1.

## DISCUSSION

This is the first meta‐analysis to evaluate the efficacy and acceptability of stimulants for the treatment of preschoolers with ADHD. Five RCTs involving 489 participants were included. Results demonstrate a significant effect of stimulants compared to placebo in change of ADHD symptoms (SMD = −0.59). Despite the concern for publication bias, this finding supports the short‐term efficacy of stimulants to reduce ADHD symptoms among preschoolers because the quality of evidence was considered moderate. Subgroup analyses did not identify significant differences, and the results remained the same after excluding studies at high risk of bias or with specific methodological characteristics (i.e., less than 2 weeks of treatment, requirement of psychotherapy prior to pharmacological treatment, and crossover design), indicating the robustness of the findings.

Although the effect size (SMD = −0.59) was smaller than previously reported for school‐age children and adolescents (SMD = −1.02, for amphetamines, −0·78, for methylphenidate) (Cortese et al., 2018), our meta‐analysis reinforces that stimulants are efficacious in the treatment of preschoolers with ADHD and may help inform treatment recommendations. These findings are particularly relevant in the context that most clinicians do not follow current treatment guidelines (Moran et al., 2019), and many preschoolers either receive medications with no evidence base or remain untreated (Danielson et al., 2017). Moreover, many barriers still limit children's access to behavioral interventions, and for many children, medication may represent a more feasible treatment.

We were unable to detect a significant effect of stimulants on all‐cause discontinuation rates. On one hand, the lack of a significant effect may be related to the small number of RCTs available, particularly considering the relatively low event rates that were observed in the included RCTs. On the other hand, the lack of a significant effect may be related to pooling studies with different dosing designs. In a recent systematic review and meta‐analyses, we showed that dose escalation adopted in flexible‐dose studies increases acceptability of medications, while the same association is not found in fixed‐dose studies which administer random doses to participants without considering their individual benefit‐to‐risk ratio of higher doses (Farhat et al., 2022). Indeed, in our analyses flexible‐dose studies (Childress et al., 2020; Sugaya et al., 2022) demonstrated increased acceptability whereas the fixed‐dose studies (Childress et al., 2022; Greenhill et al., 2006; Musten et al., 1997) demonstrated decreased acceptability. Regardless, because there are few studies available, we opted to consider the pooled evidence and downgrade the quality of evidence due to imprecision rather than focus on the subgroup analysis to avoid overinterpreting findings at this stage.

Due to the lack of data on adverse event dropout rates, it was not possible to examine stimulants' tolerability. However, results from the individual RCTs indicate that stimulants are generally safe in the short‐term for the treatment of preschoolers with ADHD. Across studies, only one serious adverse event was judged to be related to medication (i.e., a possible seizure), as described by the PATS study (Wigal et al., 2006). Most adverse events reported by the RCTs were considered mild or moderate. In all studies, except for Musten et al. (1997) (Musten et al., 1997)—which did not describe the occurrence of specific adverse events—decreased appetite, irritability, and insomnia were reported among the most frequent adverse events. Decrease in bodyweight/BMI was reported by 4 RCTs, but severe weight loss was only reported for 3 children in the PATS study. Severe insomnia was reported by 8 children, 6 of them in the PATS. Specifically, irritability/affect lability may be clinically relevant adverse events that may occur more frequently among preschoolers than in school‐aged children. It was reported as the reason for discontinuation by 9 of 14 children in the PATS study open‐label lead‐in phase (Greenhill et al., 2006) and by 3 of 8 children in the study conducted by Childress et al. (2022) (Childress et al., 2022). On the other hand, anxiety was reported in 2 studies, and severe anxiety was reported once in the PATS study (Wigal et al., 2006).

Other adverse events may warrant some attention because of potential long‐term concerns. First, increase in heart rate and blood pressure was observed across studies, except for Musten et al. (1997) (Musten et al., 1997). In the PATS study (Wigal et al., 2006) and in the MAPPA study (Sugaya et al., 2022), there was no significant difference between children using stimulants or placebo. However, Childress et al. (2020) (Childress et al., 2020) described 3 cases of hypertension and 1 case of tachycardia as possibly or probably related to the use of medication. It is possible that children exposed to stimulants at an early age may develop cardiovascular adverse events in the long‐term, for example, increased heart rate. It is important to consider that drug exposure may have different effects across developmental maturation stages. However, for individuals who started using stimulants during school‐age years, only mild increases in heart rate have been documented and there was no evidence of increased risk of hypertension over a 10‐year period (Vitiello et al., 2012). Also, a meta‐analysis of observational studies did not show a significant association between medications for ADHD and serious cardiovascular events such as stroke, myocardial infarction, or death from any cause, although a modest increase in the risk of sudden death/arrythmia were not ruled‐out (Liu et al., 2019). Second, the PATS study—the only that included a 10‐month open‐label maintenance phase—found decrements in growth rates in preschoolers on medication compared to placebo (Swanson et al., 2006). Evidence suggests that stimulants effect on height may be moderated by the level of stimulant exposure and may be attenuated by treatment discontinuation (Greenhill et al., 2020). However, more studies focusing on long‐term implications of stimulants for preschool ADHD are necessary to provide a clearer understanding of the potential hazards of stimulant use in this population.

Our study has some limitations. First, only 5 RTCs were identified (one with lisdexamfetamine, one with methylphenidate extended‐release, and three with methylphenidate immediate‐release). We were underpowered to detect significant differences in dichotomous outcomes and subgroup analyses. Hence, negative findings for subgroup analyses have to be interpreted with caution. Second, our analyses pooled data from multiple informants (i.e., parents, parent‐teachers, and clinicians). Although we were unable to detect significant differences across informants, future meta‐analyses should consider evaluating data from each informant separately as more RCTs become available. Third, due to the lack of data, it was not possible to examine stimulants' tolerability or the occurrence of specific adverse events. Fourth, we cannot discard the presence of publication bias. Although the funnel plot and Egger regression test are traditionally done with 10 or more studies, they have been previously used in meta‐analysis with 5 studies (Zhou et al., 2021). We opted to use these methods to provide the readership some visual evidence of publication bias. The findings should be interpreted with caution, but, irrespective of the limited number of RCTs, we detected a slight asymmetry in the funnel plot and the Egger regression test was significant. Regardless of statistical tests, we also note that there has been a recent increased interest in the effects of stimulants for preschoolers, with 3 of the 5 studies published in the last 3 years (Childress et al., 2020, 2022; Sugaya et al., 2022). Some of these studies also adopted a design that may maximize efficacy. For instance, the PATS (Greenhill et al., 2006) and Childress et al. (2020) (Childress et al., 2020) studies have multiple steps which may contribute to self‐selection of participants who experience the most benefit with the medication. Additional studies are required to corroborate the findings from the ones that have been included in this review. Nevertheless, the confidence of the estimate was still judged at moderate. Fifth, most studies were conducted in North America (i.e., United States and Canada), and only one study was conducted in a low‐middle income country (i.e., Brazil), limiting the generalizability of the findings to developing countries. Sixth, our analyses were restricted to stimulants' effect on ADHD symptoms and did not evaluate functional outcomes. Finally, treatment duration ranged from 1 to 8 weeks and no inference can be made about the long‐term effect and acceptability of stimulants for preschoolers.

## CONCLUSION

In conclusion, our meta‐analysis demonstrated stimulants' efficacy in reducing ADHD symptoms among preschoolers, with moderate quality of evidence. For acceptability, stimulants did not lead to an increased rate of all‐cause discontinuation rates in comparison to placebo, with very low quality of confidence on evidence. These results should be considered by clinicians when making treatment recommendations. In clinical practice, adverse events should be monitored and balanced against medication benefits.

Additional RCTs evaluating the short‐ and long‐term efficacy and safety of simulants for preschool children are still a priority in the field. Specifically, more studies evaluating long‐acting formulations of methylphenidate and amphetamines are needed. Furthermore, there is also a need for more studies that do not adopt an enrichment design, that is, select participants who were responders in an initial open‐label phase, because this design may artificially increase the effect size in favor of the medication. Future RCTs may also evaluate stimulants as first‐line intervention (i.e., without requiring prior/concomitant psychotherapy), and a flexible dosing titration may increase tolerability. Detailed reports of adverse events, including measures of blood pressure, pulse, bodyweight, and height are important, especially over longer periods of time. Future research should also fill evidence gaps including the effect of these medications on functional outcomes (e.g., school readiness, global functioning) as well potential moderators and mediators of increased/decreased effect.

## PRACTICAL GUIDANCE: CLINICAL RECOMMENDATIONS

### Assessment

Accurate diagnosis is essential and clinical assessment of preschoolers can be challenging. A discussion on this topic is beyond the scope of this review. The assessment of preschool children must include a comprehensive multi‐informant, multi‐method approach that takes into account not only current behavioral concerns, but all domains of development, home and school environment, and the level and nature of the impairment. Also, the assessment of personal and family history of cardiac diseases, and complete medical evaluation are necessary. Physical examination should include measures of height, weight, blood pressure, and pulse. Consultation from specialists (e.g., cardiologists), and complementary exams (e.g., electrocardiogram) are not routinely needed and should be required based on an individualized evaluation.

### Treatment planning

For school‐age children and adolescents with ADHD, there are numerous RCTs evaluating the effect of stimulants. Meta‐analysis demonstrated the efficacy of both methylphenidate and amphetamines. However, amphetamines increased diastolic blood pressure and were less well tolerated than placebo, while methylphenidate had better acceptability than placebo. Thus, methylphenidate has been suggested as the first‐line pharmacological treatment (Cortese et al., 2018).

In contrast, we found only 5 double‐blind RCTs evaluating the effect of stimulants for preschoolers with ADHD. The meta‐analysis demonstrated stimulants' efficacy in reducing ADHD symptoms. However, analyses were underpowered to detect significant differences in the effect of methylphenidate and amphetamines. Conversely, subgroup analyses identified differences between methylphenidate and amphetamine for acceptability, but the quality of evidence was considered very‐low. Therefore, treatment planning for preschool children with ADHD should consider both the findings and limitations of the study.

In this context, honest communication and a shared decision between clinicians and parents are recommended. Individual treatment plans should initiate with education of families and teachers, context modification and accommodations, and take into account symptom severity, functional impairment, presence of comorbidities, dysfunctional parenting practices, treatment availability, and family preferences. Discussion with families should consider both treatments' risks and benefits, and the negative consequences of ADHD.

Currently, guidelines recommend psychosocial interventions as first‐line treatment for preschoolers with ADHD (Cortese, 2020). However, availability of evidence‐based therapies is very limited across the world, especially in settings with limited resources. Also, meta‐analysis does not support the efficacy of BPT in reducing ADHD symptoms when outcomes were rated by blinded evaluators (Rimestad et al., 2019). In the MAPPA study (Sugaya et al., 2022), a multi‐arm, randomized, double‐blind, placebo‐ and sham BPT‐controlled clinical trial comparing methylphenidate and BPT over a control group, BPT had no significant effect over reduction of symptoms in comparison to control group, despite an effect size of −0.44 (95% CI −0.89, 0.003), and produced an improved global functioning. In addition, most guidelines were published before 2020 and, at that point, there were only 2 double‐blind RCTs evaluating the use of stimulants for preschoolers with ADHD (Greenhill et al., 2006; Musten et al., 1997). Since then, 3 additional double‐blind RCTs were published showing stimulants' efficacy in reducing ADHD symptoms when compared to placebo (Childress et al., 2020, 2022; Sugaya et al., 2022) and current meta‐analysis supports the efficacy of stimulants with moderate quality of evidence.

Thus, we argue that BPT must be indicated particularly for parents whose children present comorbid oppositional defiant disorder or conduct problems (Groenman et al., 2021), or for parents who display significant dysfunctional practices to manage their children's behavior (Rimestad et al., 2019), and in settings where parents can have access to it. Stimulants should also be considered without the requirement of a previous trial of BPT for treatment of preschoolers with ADHD especially for children >4 years of age with moderate or severe symptoms; when there is limited access to BPT; and when the pharmacological treatment is the family preference. Considering that most RCTs evaluated the use of methylphenidate immediate‐release, it may be the medication of choice. Long‐acting methylphenidate formulation and lisdexamfetamine can also be considered. A slow titration regimen with frequent re‐assessments is recommended to optimize treatment and improve tolerability. Treatment monitoring should include a systematic assessment of symptoms, functional impairment, and adverse events including decreased appetite, irritability, and insomnia, as well as measures of height, bodyweight, pulse, and blood pressure. Trials of discontinuation should be actively considered and discussed with families, especially if there is remission of symptoms over 6–12 months of follow‐up.

To date, there is no clinical trial evaluating the combination of stimulants and behavioral interventions for preschoolers with ADHD. It is hypothesized that these two treatments have different mechanisms and could be used to target different areas of impairment. In the MAPPA study (Sugaya et al., 2022), secondary outcome analyses showed that methylphenidate was associated with improvement in cognitive measures of attention while BPT was associated with improvement in irritability. Therefore, specific groups may particularly benefit from a multimodal approach as reported by the Multimodal Treatment of ADHD Study (MTA). In the MTA, school‐age children with comorbid anxiety and disruptive behavior disorder had a greater response to the combined treatment (Jensen et al., 2001). Future studies with preschoolers evaluating the efficacy of combined treatment will be important to guide treatment recommendations.

For children that do not tolerate the use of stimulants, alpha‐agonists could be considered with caution. Indeed, alpha‐agonists have been commonly used among this population, both in isolation or in combination with stimulants (Panther et al., 2017). However, clinicians should take into account that there is no double‐blind RCT evaluating the use of alpha‐agonists for preschool children with ADHD, and its use has been supported by case reports (Lee, 1997) and a retrospective chart review (Harstad et al., 2021). Based on data from studies conducted with older children, prior to the use of alpha‐agonists, clinicians should assess the occurrence of symptoms of hypotension (e.g., dizziness), cardiac conditions, and family history of QTc prolongation. Electrocardiogram and consultation from specialists should be considered for specific individuals (Hirota et al., 2014). To date, there is only one double‐blind RCT that compared atomoxetine and placebo for the treatment of children aged 5–6 years (Kratochvil et al., 2011). Although results showed a reduction of ADHD symptoms, most children remained moderately to severely ill at the completion of the study, and they were more likely to have decreased appetite, gastrointestinal upset, and sedation.

Antipsychotics are widely prescribed for young children across the globe (Sultan et al., 2019) but current evidence does not support ADHD as an indication. There is one small double‐blind RCT comparing risperidone and methylphenidate for children with 3–6 years, with no difference in response between groups (Arabgol et al., 2015). The long‐term metabolic adverse events that have already been associated with the use of antipsychotics, including weight gain, hyperlipidemia, diabetes, and negative impact on bone metabolism are concerning (Correll, 2009). One exception are school‐age children with ADHD and comorbid disruptive behavior disorders or severe aggressiveness. Studies with this population showed that adding risperidone, after behavioral intervention and stimulant optimization, reduced aggressiveness, ADHD, and ODD symptoms (Blader et al., 2021). Importantly, before adding a second medication targeting disruptive behaviors, it is fundamental to optimize the stimulant dose (Blader et al., 2021).

Irrespective of the chosen treatment, clinicians should be aware that there is limited evidence regarding the risks and benefits of long‐term treatment. Thus, careful and continuous clinical assessment of symptoms, global functioning, and adverse events is mandatory, as well as periodic revision of diagnosis, new arising comorbidities and family environment. In the future, rigorous RCTs with long‐term follow‐up and the identification of clinical predictors of response and adverse events may be helpful to guide treatment decisions.

## AUTHOR CONTRIBUTIONS

**Luisa S. Sugaya:** Conceptualization; Writing – original draft; Writing – review and editing. **Luis C. Farhat:** Conceptualization; Data curation; Formal analysis; Methodology; Writing – original draft; Writing – review and editing. **Pietro Califano:** Data curation; Writing – review and editing. **Guilherme V. Polanczyk:** Conceptualization; Funding acquisition; Methodology; Supervision; Writing – original draft; Writing – review and editing.

## CONFLICT OF INTEREST STATEMENT

GVP, in the last 3 years, has been a consultant, member of advisory board, and/or speaker for Aché, Abbott, Medice, Novo Nordisk, and Takeda; he has received royalties from Editora Manole. He is also a Joint Editor for JCPP *Advances*. The remaining authors have declared they have no potential or competing conflicts of interest.

### OPEN RESEARCH BADGES

This article has been awarded <Open Data, Open Material, Preregistered> badges. All materials and data are publicly accessible via the Open Science Framework at (https://osf.io/98wxt/). Learn more about the Open Practices badges from the Center for Open Science: https://osf.io/tvyxz/wiki

## ETHICAL CONSIDERATIONS

An ethics statement is not applicable to this paper.

## PROTOCOL REGISTRATION

PROSPERO CRD42022348597.

## Supporting information

Supporting Information S1

## Data Availability

The data that supports the findings of this study are available at https://osf.io/98wxt/.
