# Supplementary material for: Efficacy of stimulants for preschool attention‐deficit/hyperactivity disorder: A systematic review and meta‐analysis
Source: JCPP Adv. 2023 Feb 25;3(3):e12146. doi: 10.1002/jcv2.12146 (PMC10501696; doi:10.1002/jcv2.12146)
Supplement: Supplementary file 1 — Supporting Information S1 [file JCV2-3-e12146-s001.docx]

**Supporting Information**

| **Table of Contents** | **PAGE** |
| --- | --- |
| PRISMA Checklist | 2 |
| Search Strategy | 5 |
| Domain-level risk of bias assessment for each study | 6 |
| Supplementary figures | 10 |
| **Figure S1.** Forest plot for efficacy in sensitivity analysis excluding studies rated at high risk of bias. | 10 |
| **Figure S2.** Forest plot for efficacy in sensitivity analysis excluding studies which lasted for less than 2 weeks. | 10 |
| **Figure S3.** Forest plot for efficacy in sensitivity analysis excluding studies which required participants to receive psychotherapy prior to pharmacological treatment. | 10 |
| **Figure S4.** Forest plot for efficacy in sensitivity analysis excluding crossover studies. | 11 |
| **Figure S5.** Funnel plot for efficacy | 11 |
| Domain-level quality of evidence assessment for each outcome | 12 |

**PRISMA CHECKLIST**

| **Section and Topic** | **Item #** | **Checklist item** | **Location where item is reported** |
| --- | --- | --- | --- |
| **TITLE** | | |  |
| Title | 1 | Identify the report as a systematic review. | p 1 |
| **ABSTRACT** | | |  |
| Abstract | 2 | See the PRISMA 2020 for Abstracts checklist. | p 3-4 |
| **INTRODUCTION** | | |  |
| Rationale | 3 | Describe the rationale for the review in the context of existing knowledge. | pp 6-7 |
| Objectives | 4 | Provide an explicit statement of the objective(s) or question(s) the review addresses. | p 7 |
| **METHODS** | | |  |
| Eligibility criteria | 5 | Specify the inclusion and exclusion criteria for the review and how studies were grouped for the syntheses. | pp 7-8 |
| Information sources | 6 | Specify all databases, registers, websites, organisations, reference lists and other sources searched or consulted to identify studies. Specify the date when each source was last searched or consulted. | p 8 |
| Search strategy | 7 | Present the full search strategies for all databases, registers and websites, including any filters and limits used. | Supp 5 |
| Selection process | 8 | Specify the methods used to decide whether a study met the inclusion criteria of the review, including how many reviewers screened each record and each report retrieved, whether they worked independently, and if applicable, details of automation tools used in the process. | pp 8-9 |
| Data collection process | 9 | Specify the methods used to collect data from reports, including how many reviewers collected data from each report, whether they worked independently, any processes for obtaining or confirming data from study investigators, and if applicable, details of automation tools used in the process. | pp 8-10 |
| Data items | 10a | List and define all outcomes for which data were sought. Specify whether all results that were compatible with each outcome domain in each study were sought (e.g. for all measures, time points, analyses), and if not, the methods used to decide which results to collect. | pp 9-10 |
|  | 10b | List and define all other variables for which data were sought (e.g. participant and intervention characteristics, funding sources). Describe any assumptions made about any missing or unclear information. | pp 9-10 |
| Study risk of bias assessment | 11 | Specify the methods used to assess risk of bias in the included studies, including details of the tool(s) used, how many reviewers assessed each study and whether they worked independently, and if applicable, details of automation tools used in the process. | pp 9,11 |
| Effect measures | 12 | Specify for each outcome the effect measure(s) (e.g. risk ratio, mean difference) used in the synthesis or presentation of results. | p 10 |
| Synthesis methods | 13a | Describe the processes used to decide which studies were eligible for each synthesis (e.g. tabulating the study intervention characteristics and comparing against the planned groups for each synthesis (item #5)). | pp 9-10 |
|  | 13b | Describe any methods required to prepare the data for presentation or synthesis, such as handling of missing summary statistics, or data conversions. | pp 10-11 |
|  | 13c | Describe any methods used to tabulate or visually display results of individual studies and syntheses. | p 11 |
|  | 13d | Describe any methods used to synthesize results and provide a rationale for the choice(s). If meta-analysis was performed, describe the model(s), method(s) to identify the presence and extent of statistical heterogeneity, and software package(s) used. | pp 10-11 |
|  | 13e | Describe any methods used to explore possible causes of heterogeneity among study results (e.g. subgroup analysis, meta-regression). | p 10 |
|  | 13f | Describe any sensitivity analyses conducted to assess robustness of the synthesized results. | p 11 |
| Reporting bias assessment | 14 | Describe any methods used to assess risk of bias due to missing results in a synthesis (arising from reporting biases). | p 11 |
| Certainty assessment | 15 | Describe any methods used to assess certainty (or confidence) in the body of evidence for an outcome. | p 11 |
| **RESULTS** | | |  |
| Study selection | 16a | Describe the results of the search and selection process, from the number of records identified in the search to the number of studies included in the review, ideally using a flow diagram. | pp 11-12 |
|  | 16b | Cite studies that might appear to meet the inclusion criteria, but which were excluded, and explain why they were excluded. | Figure 1 |
| Study characteristics | 17 | Cite each included study and present its characteristics. | pp 11-12 |
| Risk of bias in studies | 18 | Present assessments of risk of bias for each included study. | Supp 7-9 |
| Results of individual studies | 19 | For all outcomes, present, for each study: (a) summary statistics for each group (where appropriate) and (b) an effect estimate and its precision (e.g. confidence/credible interval), ideally using structured tables or plots. | Table 1 |
| Results of syntheses | 20a | For each synthesis, briefly summarise the characteristics and risk of bias among contributing studies. | Supp 12-13 |
|  | 20b | Present results of all statistical syntheses conducted. If meta-analysis was done, present for each the summary estimate and its precision (e.g. confidence/credible interval) and measures of statistical heterogeneity. If comparing groups, describe the direction of the effect. | pp 12-13 |
|  | 20c | Present results of all investigations of possible causes of heterogeneity among study results. | pp 12-13 |
|  | 20d | Present results of all sensitivity analyses conducted to assess the robustness of the synthesized results. | pp 12-13 |
| Reporting biases | 21 | Present assessments of risk of bias due to missing results (arising from reporting biases) for each synthesis assessed. | pp 12- 13 |
| Certainty of evidence | 22 | Present assessments of certainty (or confidence) in the body of evidence for each outcome assessed. | pp 12- 13 |
| **DISCUSSION** | | |  |
| Discussion | 23a | Provide a general interpretation of the results in the context of other evidence. | pp 13-16 |
|  | 23b | Discuss any limitations of the evidence included in the review. | pp 14-16 |
|  | 23c | Discuss any limitations of the review processes used. | pp 15-16 |
|  | 23d | Discuss implications of the results for practice, policy, and future research. | pp 17-18 |
| **OTHER INFORMATION** | | |  |
| Registration and protocol | 24a | Provide registration information for the review, including register name and registration number, or state that the review was not registered. | p 8 |
|  | 24b | Indicate where the review protocol can be accessed, or state that a protocol was not prepared. | p 8 |
|  | 24c | Describe and explain any amendments to information provided at registration or in the protocol. | p 10 |
| Support | 25 | Describe sources of financial or non-financial support for the review, and the role of the funders or sponsors in the review. | p 1 |
| Competing interests | 26 | Declare any competing interests of review authors. | p 1 |
| Availability of data, code and other materials | 27 | Report which of the following are publicly available and where they can be found: template data collection forms; data extracted from included studies; data used for all analyses; analytic code; any other materials used in the review. | p 1 |

**SEARCH STRATEGY**

**PubMed**

("Attention Deficit Disorder with Hyperactivity"[Mesh] OR adhd[tiab] OR "attention deficit*"[tiab]) AND ("Methylphenidate"[Mesh] OR "Dexmethylphenidate"[Mesh] OR Focalin[tiab] OR Dexmethylphenidate[tiab] OR Methylphenidate[tiab] OR Biphentin[tiab] OR Concerta[tiab] OR Daytrana[tiab] OR Methylphenidate[tiab] OR Equasym[tiab] OR Methylin[tiab] OR Ritalin*[tiab] OR Medikinet[tiab] OR Metadate[tiab] OR Quillivant[tiab] OR "Amphetamines"[Mesh] OR Adderall[tiab] OR Amphetamine[tiab] OR Amfetamine[tiab] OR Dexamfetamine[tiab] OR Dexamphetamine[tiab] OR Dexedrine[tiab] OR Dextroamphetamine[tiab] OR DextroStat[tiab] OR Elvanse[tiab] OR Lisdexamfetamine[tiab] OR Vyvanse[tiab]) AND (preschool* OR toddler*) AND (randomized controlled trial[pt] OR controlled clinical trial[pt] OR randomized[tiab] OR placebo[tiab] OR clinical trials as topic[mesh:noexp] OR randomly[tiab] OR trial[ti]) NOT (animals[mh] NOT humans[mh])

**Embase**

("Attention Deficit Disorder with Hyperactivity" OR adhd OR "attention deficit*") AND ("Methylphenidate" OR "Dexmethylphenidate" OR Focalin OR Dexmethylphenidate OR Methylphenidate OR Biphentin OR Concerta OR Daytrana OR Methylphenidate OR Equasym OR Methylin OR Ritalin* OR Medikinet OR Metadate OR Quillivant OR "Amphetamines" OR Adderall OR Amphetamine OR Amfetamine OR Dexamfetamine OR Dexamphetamine OR Dexedrine OR Dextroamphetamine OR DextroStat OR Elvanse OR Lisdexamfetamine OR Vyvanse) AND (preschool* OR toddler*) AND (randomized controlled trial OR controlled clinical trial OR randomized OR placebo OR randomly OR trial) NOT (animals NOT humans)

**CENTRAL**

(("Attention Deficit Disorder with Hyperactivity" OR adhd OR "attention deficit*")) AND (("Methylphenidate" OR "Dexmethylphenidate" OR Focalin OR Dexmethylphenidate OR Methylphenidate OR Biphentin OR Concerta OR Daytrana OR Methylphenidate OR Equasym OR Methylin OR Ritalin* OR Medikinet OR Metadate OR Quillivant OR "Amphetamines" OR Adderall OR Amphetamine OR Amfetamine OR Dexamfetamine OR Dexamphetamine OR Dexedrine OR Dextroamphetamine OR DextroStat OR Elvanse OR Lisdexamfetamine OR Vyvanse)) AND ((preschool* OR toddler*)) AND ((randomized controlled trial OR controlled clinical trial OR randomized OR placebo OR randomly OR trial)):ti,ab,kw

**WHO ICTRP**

("Attention Deficit Disorder with Hyperactivity" OR adhd OR "attention deficit*") AND (preschool* OR toddler*)

**DOMAIN-LEVEL RISK OF BIAS ASSESSMENT FOR EACH STUDY**

**Childress_2020**

| **Domain** | **Judgment** | **Justification** |
| --- | --- | --- |
| Randomisation process | Low | “Children were randomized in a 1:1 ratio through a computer-generated randomization schedule to either continue their optimized dose or receive matching placebo” (Journal article, page 60).  There are no specific pieces of information about how allocation sequence was concealed during the study. However, the investigator confirmed sites were not aware of the sequence (correspondence through email)  Baseline characteristics were relatively similar across treatment groups. (Journal article, Table 1) |
| Deviations from interventions | Low | A matching placebo was used.  There are no specific pieces of information about blinding of therapists. However, the investigator confirmed site personnel did not know what treatments subjects received (correspondence through email).  “Several populations were defined for purposes of data analysis: (3) ITT-efficacy evaluable (ITT-E) population, including all children in the ITT population who completed ADHD-RS-IV assessments at the end of the open-label Phase 4 (DB phase baseline) and had at least one postbaseline ADHD-RS-IV assessment” (journal article, page 61) |
| Missing outcome data | Low | Only 4 participants discontinued the study during the double-blinded phase. (Journal article, Figure 2) |
| Outcome measurement | Low | There are no specific pieces of information about blinding of investigators/outcome assessors. However, the investigator confirmed site personnel did not know what treatments subjects received (correspondence through email) |
| Outcome selection | Low | “The primary efficacy measure is the comparison of the two treatment groups (optimized dose vs placebo) using the change in ADHD-RS-IV Total Score during the double-blind phase, i.e., the change from end of open label phase to end of double-blind phase” (protocol, available in ClinicalTrials.gov) |

**Childress_2022**

| **Domain** | **Judgment** | **Justification** |
| --- | --- | --- |
| Randomisation process | Low | “In this double-blind study, treatment assignments were determined using a randomization schedule, with each treatment assigned by an interactive Web response system (IWRS)” (Journal article, page 3).  Baseline characteristics were relatively similar across treatment groups. (Journal article, Table 1) |
| Deviations from interventions | Low | “To protect study blinding, LDX and PBO capsules appeared identical” (journal article, page 3).  There were numerous protocol deviations, however analyses followed a modified intent-to-treat principle: “Statistical analysis of efficacy compared the pooled LDX doses (i.e., the 10-,20-, and 30-mg doses) with PBO, and included all randomized participants receiving ≥ 1 dose of LDX and having ≥ 1 postbaseline ADHD-RS-IV-PS-TS assessment”.  Participants from the 5 mg arm were excluded from efficacy analysis, but this was pre-specified in the statistical analysis plan “Specifically, these analyses will compare placebo and pooled SPD489 10, 20, 30 mg dose strengths together, excluding the 5 mg arm” (statistical analysis plan, page 26, available in ClinicalTrials.gov) |
| Missing outcome data | Some concerns | There were some discontinuations (40, 25%) and those were relatively imbalanced across treatment arms (PBO 8, 17%; LDX 32, 28%). However, discontinuations due to adverse events were relatively balanced across groups (PBO 2, 4%; LDX 5%). |
| Outcome measurement | Low | Investigators were blinded to treatment assignments. |
| Outcome selection | Low | “The primary efficacy endpoint is defined as the change from baseline in clinician-administered ADHD-RS-IV Preschool Version Total Score at Visit 6 (Week 6)” (statistical analysis plan, page 26). |

**Greenhill_2006**

| **Domain** | **Judgment** | **Justification** |
| --- | --- | --- |
| Randomisation process | Low | “Randomization was done centrally at the coordinating site using a computerized stratified randomization, 1:1:1:1 starting dose allocation ratio, using a randomized, balanced, crossover protocol designed to avoid order effects. A second randomization to active MPH or to placebo was performed before entering the parallel-design, placebo-controlled phase” (Journal article, page 1286) |
| Deviations from interventions | Low | “Except in emergencies, clinicians remained blind to the dose sequences.” (Journal article, page 1286).  “Subjects were randomized to one of five sequences of four different MPH doses (1.25, 2.5, 5, 7.5 mg) and placebo administered t.i.d. in identical capsules for 1 week each” (Journal article, page 1286).  “All analyses were run using the intent-to-treat principle (i.e., each observation obtained for the child was used in the analysis including those from children who entered each of the two phases under consideration and did not complete the phase”. |
| Missing outcome data | High | There were considerable discontinuations (36, 32%) and those were imbalanced across treatment arms (PBO 24, 45%; MPH 9, 15%) |
| Outcome measurement | Low | Parents and teachers were blinded to treatment assignment |
| Outcome selection | Some concerns | Change in ADHD symptoms considering the primary efficacy composite SNAP as a continuous measure was a *post hoc* analysis. “In an additional post hoc, intent-to-treat, last observation carried forward analysis, we used the primary efficacy composite SNAP rating of the parallel phase as a continuous measure to supplement the categorical approach cited above”. (Journal article, page 1290). |

**Musten_1997**

| **Domain** | **Judgment** | **Justification** |
| --- | --- | --- |
| Randomisation process | Low | “Treatment was presented in a fully randomized order prepared by the hospital’s pharmacy department” (Journal article, page 1408). |
| Deviations from interventions | Low | “Two doses (0.3 mg/kg and 0.5 mg/kg) of MPH and a lactose placebo were prepared by the pharmacy at the Children’s Hospital of Eastern Ontario to the nearest 2.5 mg and placed in orange gelatin capsules (size 16, Ely Lilly Company) to disguise the taste differences between placebo and the two doses.” (Journal article, page 1408)  “all subjects, research personnel, and medical personnel were unaware of the order” (Journal article, page 1408) |
| Missing outcome data | Some concerns | “A final total of 41 children participated in the medication phase after parents received feedback and gave informed consent for medication. Of these, 31 children completed the treatment regimen, 4 children withdrew from treatment, and 6 children did not have completed assessment protocols (questionnaires) after one or all of the treatment phases”. |
| Outcome measurement | Low | Parents were blinded to treatment. |
| Outcome selection | Low | We were unable to locate a protocol. However, the CPRS was used, and the hyperactivity index was reported – those procedures were considered expected in the field at the time of the study. |

**Sugaya et al., 2022**

| **Domain** | **Judgment** | **Justification** |
| --- | --- | --- |
| Randomisation process | Low | “The randomization scheme was generated by an independent research manager with experience in the execution of clinical trials and no involvement in this trial using a permuted block randomization procedure with equal allocation and 5 blocks with N = 30 (<https://www.randomization.com>). Three additional participants that had initiated the pre-trial assessment before the inclusion of the 150th participant were considered eligible and were randomized. For their inclusion, an identical randomization scheme was generated, except that the block size was 3.” (Supporting file, page 3) |
| Deviations from interventions | Low | “Parents, teachers, child and adolescent psychiatrists that conducted child’s initial assessment and clinical evaluations during the trial, research assistants, and independent evaluators remained fully blinded. Only the study pharmacist was unblinded to medication treatment” (Supporting file, page 3)  “Placebo was corn flour only, encapsulated in capsules identical to the MPH in color, size, and weight.” (Journal article, page 9) |
| Missing outcome data | Low | There were few discontinuation rates in the study. |
| Outcome measurement | Low | “The first primary outcome was ADHD symptoms measured by the SNAP-IV scale, which was rated by a blinded independent evaluator based on parental interview and completed independently by teachers who were also blinded to treatment conditions” (Journal article, page 11) |
| Outcome selection | Low | The SNAP was the primary efficacy endpoint in ClinicalTrials.gov |

**SUPPLEMENTARY FIGURES**

**
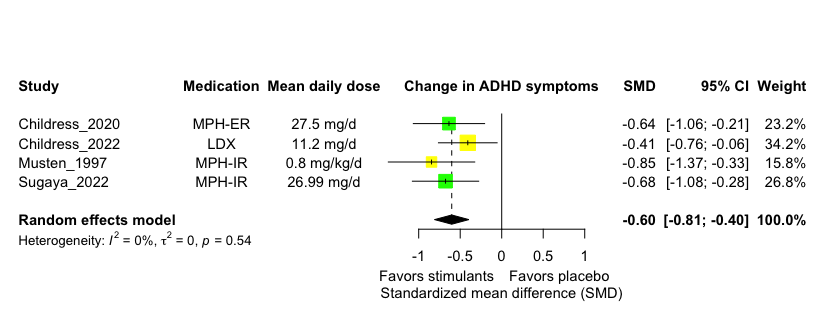
**

**Figure S1**. Forest plot for efficacy in sensitivity analysis excluding studies rated at high risk of bias.

LDX, lisdexamfetamine; MPH-ER, methylphenidate Extended-release; MPH-IR, methylphenidate immediate-release; SMD, standardized mean difference. Risk of bias: green, low risk; yellow, some concern.

**
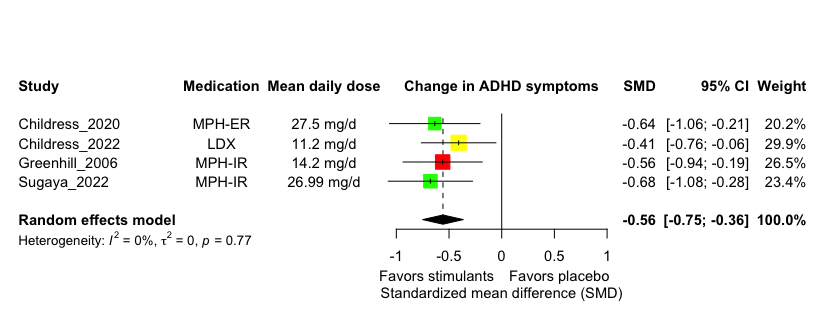
**

**Figure S2**. Forest plot for efficacy in sensitivity analysis excluding studies which lasted for less than 2 weeks.

LDX, lisdexamfetamine; MPH-ER, methylphenidate Extended-release; MPH-IR, methylphenidate immediate-release; SMD, standardized mean difference. Risk of bias: green, low risk; yellow, some concern; red, high risk

**
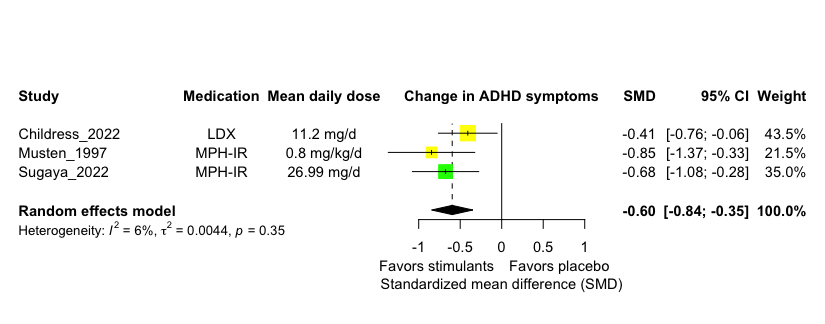
**

**Figure S3**. Forest plot for efficacy in sensitivity analysis excluding studies which required participants to receive psychotherapy prior to pharmacological treatment.

LDX, lisdexamfetamine; MPH-IR, methylphenidate immediate-release; SMD, standardized mean difference Risk of bias: green, low risk; yellow, some concern.

**
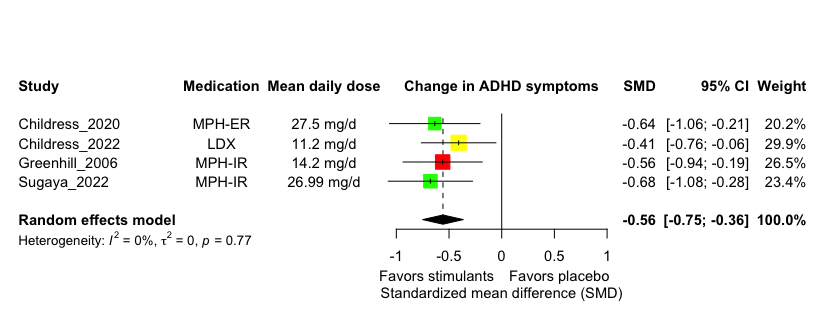
**

**Figure S4**. Forest plot for efficacy in sensitivity analysis excluding crossover studies.

LDX, lisdexamfetamine; MPH-ER, methylphenidate Extended-release; MPH-IR, methylphenidate immediate-release; SMD, standardized mean difference. Risk of bias: green, low risk; yellow, some concern; red, high risk

**
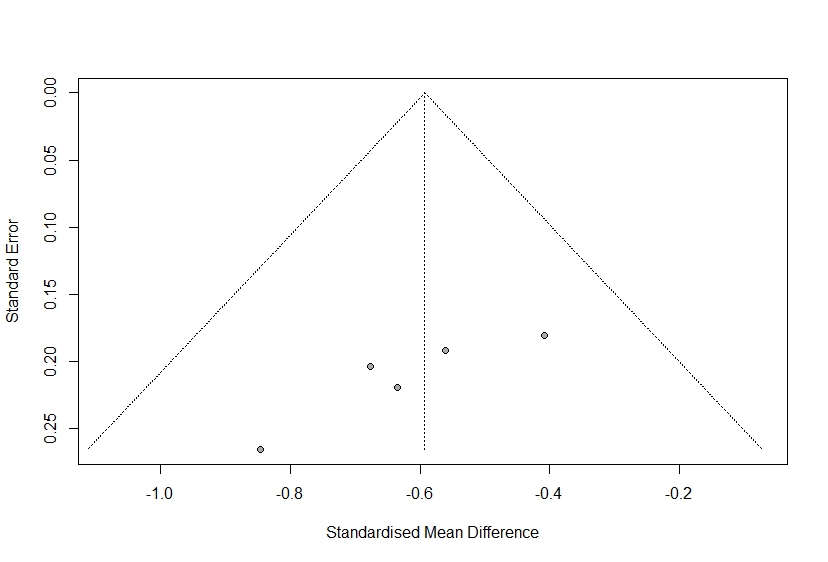
**

**Figure S5**. Funnel plot for efficacy

**DOMAIN-LEVEL QUALITY OF EVIDENCE ASSESSMENT FOR EACH OUTCOME**

**Efficacy**

| **Domain** | **Judgment** | **Justification** |
| --- | --- | --- |
| Study limitations | No serious limitation, do not downgrade | Most information came from studies at low or at some concerns of risk of bias. Therefore, we considered that potential limitations were unlikely to lower confidence in the estimate of effect following recommendations from GRADE. |
| Inconsistency | No serious limitation, do not downgrade | There was not evidence of statistical heterogeneity. Visual inspection of funnel plots did not indicate considerable between-study variability. |
| Imprecision | No serious limitation, do not downgrade | Considering α 0.05, β 0.2, a mean difference between treatment groups 5.94, a standard deviation of 10.62 and one-sided type I error, the calculated sample size was 82 (41 in each treatment group). Hence, our meta-analysis was powered to detect a Cohen d of 0.56.  Additionally, the 95% confidence interval does not overlap with the null effect. |
| Indirectness | No serious limitation, do not downgrade | Our strict eligibility criteria ensured our population was directly relevant to our research question (individuals in preschool with DSM ADHD). Two studies (Childress_2020, Greenhill_2006) individuals were responders to the medication that was administered in the RDBCT, which may limit generalizability. However, we opted not to downgrade the quality of evidence because most studies information came from studies in which individuals were not originally considered responders.  Also, our data concerns the short-term (median 2 weeks) and only evaluates improvement in ADHD symptom severity. Although we recognize these are important limitations from our findings, we opted not to downgrade quality of evidence because those were also characteristics of our research question. |
| Publication bias | Serious limitation, downgraded one level | We opted to downgrade one level quality of evidence due to publication bias. There was statistical evidence of publication bias and funnel plot asymmetry. Besides, most studies have been relatively recent, and early publications may be more likely to report positive results. |

**Acceptability**

| **Domain** | **Judgment** | **Justification** |
| --- | --- | --- |
| Study limitations | No serious limitation, do not downgrade | We opted not do downgrade quality of evidence because discontinuation rates are directly related to risk of bias assessments (missing outcome data). |
| Inconsistency | Serious limitation, downgraded one level | There was evidence of statistical inconsistency. Subgroup analysis stratified by compound was able to resolve heterogeneity, likely because it separated fixed-dose and flexible-dose studies. Yet, because subgroup analyses may be spurious, particularly with so few studies, we opted not to use the subgroup analysis and downgrade quality of evidence one level. |
| Imprecision | Very serious limitation, downgraded two levels | We had a small sample size and were underpowered to detect differences in dichotomous outcomes. Additionally, the 95% CI of our estimate crossed the threshold for null effect while also excluding important benefit AND important harm (0.8, 1.25) |
| Indirectness | Serious limitation, downgraded one level | A fixed-dose study provided most of the evidence (weight 37.2%) but it may not provide a representative picture of the stimulants in routine practice. |
| Publication bias | No serious limitation, do not downgrade | We opted not to downgrade quality of evidence. Although it is possible that studies with high discontinuation rates would not be published, at the same time dropout rates are not typical outcomes in RDBCTs and therefore may be less influenced by editorial preferences. |
